# Competitive suppression and release in artemisinin-resistant *Plasmodium falciparum* field isolates

**DOI:** 10.1093/emph/eoag009

**Published:** 2026-05-07

**Authors:** Katelyn Vendrely Brenneman, Tarrick Qahash, Katherine A Beckman, François H Nosten, Michael T Ferdig

**Affiliations:** Eck Institute for Global Health, University of Notre Dame, Notre Dame, IN, USA; Eck Institute for Global Health, University of Notre Dame, Notre Dame, IN, USA; Eck Institute for Global Health, University of Notre Dame, Notre Dame, IN, USA; Shoklo Malaria Research Unit, Mahidol-Oxford Tropical Medicine Research Unit, Mahidol University, Mae Sot, Thailand; Centre for Tropical Medicine and Global Health, Nuffield Department of Medicine, University of Oxford, Oxford, UK; Eck Institute for Global Health, University of Notre Dame, Notre Dame, IN, USA

**Keywords:** fitness, malaria, competitive suppression, competitive release, evolution of resistance

## Abstract

**Background and objectives:**

Infection with multiple genetically distinct *Plasmodium falciparum* parasites is common in human malaria cases. Within an infected patient, a particular genotype may thrive, influencing its chance of transmission. Competitive growth assays provide a quantitative index to assess relative dynamic fitness disparities among co-infecting parasites across experimentally controlled, physiologically relevant conditions to test specific hypotheses. The ability to assess the relative competitive fitness of parasite genotypes present in patient-derived samples will be particularly valuable to track the spread of partial artemisinin (ART) resistance now emerging in Africa.

**Methodology:**

Pairwise competitive growth outcomes of genetically distinct ART-resistant (ART-R) and ART-sensitive clones isolated from the Thailand-Myanmar border were evaluated after perturbation with dihydroartemisinin. Fluorescent labeled microsatellite markers were used to measure the relative growth densities of each competing parasite. Resistant and susceptible clones were mixed and grown alone in the presence and absence of drug to determine dynamic fitness relationships among the co-infecting parasites.

**Results:**

ART-R strains demonstrate a competitive advantage when grown in the presence of a high drug dose. However, the loss of this advantage varies in the absence of drug, producing a range of fitness phenotypes for sensitive and resistant strains.

**Conclusions and implications:**

Not surprisingly, drug-resistant parasites outcompete susceptible parasites under drug; however, additional variables influence whether resistant parasites ultimately prevail when drug is removed, suggesting independent or interacting genetic mechanisms. Knowing relative fitness advantages and costs of specific genotypes can inform how they will spread and evolve in a competitive environment, directing novel intervention strategies.

**Lay Summary:**

Malaria infections often contain both drug-resistant and drug-sensitive parasites. Parasites were co-grown with and without antimalarials and competitive outcomes varied: sometimes resistant parasites won, sometimes sensitives parasites won, and in one case, the winner changed with antimalarial treatment. Drug-resistant parasite success depends on competing parasites, complicating the prediction of resistance spread.

## INTRODUCTION

Infections by multiple genetically distinct *Plasmodium falciparum* parasites are common in cases of human malaria, especially in high-transmission settings where polygenomic infections can contain up to seven different *P. falciparum* parasite strains [1, 2]. Complexity of infection (COI) is a standard metric for the number of genetically distinct *P. falciparum* clones in a polygenomic infection and can be an indirect measure for transmission intensity [3, 4]. Polygenomic infections occur from the injection of multiple sporozoites representing multiple genotypes into a single host either from a single mosquito feeding (co-infection) or from multiple mosquito feedings (superinfection) [5, 6]. Polygenomic infections can alter the course of the infection, and within-host competition may lead to higher disease severity [7, 8]. Studies of *Plasmodium* species suggest that parasites can recognize genetically dissimilar parasites and adjust their behavior via kin discrimination [9]. Parasites also appear to communicate through quorum sensing using small signal molecules to trigger changes in gene expression in response to changes in population density [10, 11], which is also commonly seen in microbial communities and unicellular parasites [12, 13]. Red blood cell (RBC) exosome-like vesicles that are able to deliver molecules and genes may also enable communication between *Plasmodium* parasites in a population [14, 15]. These interactions and communications likely influence virulence, transmission, competition, and evolution of parasite communities.

**Figure 1:**
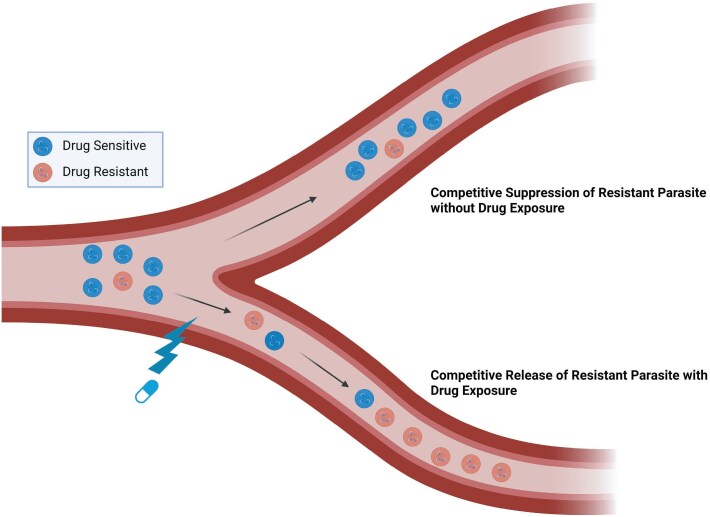
**Schematic of competitive suppression and release for parasites**; for a polyclonal infection with both drug-resistant and drug-sensitive parasites (top) without drug pressure, the drug-resistant parasite remains at the same low frequency due to intense competition from drug-sensitive parasites (competitive suppression of the drug-resistant parasite), especially when there is a fitness cost associated with drug resistance; (bottom) with drug pressure, drug-sensitive parasites are removed from the population, allowing the resistant parasite to expand to fill the niche left by the sensitive parasites, resulting in competitive release of the resistant parasite.

Resistance mutations often confer fitness advantages in the presence of drug, but they incur fitness costs in the absence of drug pressure [16]. However, fitness costs may change, especially as parasites gain compensatory mutations (i.e. mutations that enhance higher levels of resistance or compensate for fitness costs [17]). Without drug pressure in the population, drug-resistant parasites rarely emerge because there is intense competition from drug-sensitive competitors (known as competitive suppression), especially when there is a large fitness cost or an increased resource requirement associated with resistance [18–20]. Drug treatment of a polygenomic infection containing both drug-resistant and drug-sensitive parasites can remove the drug-sensitive competitors, allowing resistant parasites to expand to fill the niche left by the sensitive competitors. Drug treatment removes the competitive suppression, resulting in competitive release of the resistant parasite [21–25]. Competitive release can lead to facilitation, where drug-resistant parasites achieve higher densities than they would in a single clone (clonal) infection due to the clearance of the drug-sensitive competitors in the polygenomic infection [24]. The removal of drug-susceptible competitors using drug treatment can greatly enhance the chances of drug-resistant parasites emerging, overcoming fitness costs, and spreading in the population (Fig. 1), especially in high-COI, high-transmission settings.

Competitive growth assays are a valuable indicator of the fitness costs of drug resistance in pathogens such as HIV, fungi, and bacteria [26, 27]. For *P. falciparum*, fitness costs have been associated with artemisinin (ART)-resistance, but the complexity of the genetic determinants of resistance and its effect on competitive fitness in the absence of drug has only recently begun to be explored [28–30]. Clinically, ART-resistance is defined as delayed parasite clearance half-life in patients (>5 h) [30]. Single point mutations in the propeller domain of *kelch13* have been associated with delayed clearance and has become the molecular marker of ART-resistance [31, 32]. Many different single nucleotide polymorphisms (SNPs) are predictive of delayed parasite clearance, with the C580Y SNP being the most prevalent along the Thailand-Cambodia border [31, 32]. Although there is an association between resistance and *kelch13* mutations, parasites have been isolated from patients that exhibit delayed parasite clearance when treated with ART but lack *kelch13* mutations [33], suggesting that genes other than *kelch13* are also associated with resistance to ART.

In this study, competitive growth was assessed as an index for blood stage fitness between natural isolates with different genetic variants using a previously described *in vitro* 96-well plate method to quantitatively measure head-to-head competitive outcomes [30]. Fluorescent-labeled microsatellite markers were used to estimate the relative abundance of each parasite genome throughout the co-growth period of 64 days. To understand the fitness of ART-R parasites and the transmission potential of specific resistant variants, the competitive fitness of ART-R isolates with and without *kelch13-*associated resistance must be investigated. Findings related to the fitness costs of ART-resistance will facilitate a deeper understanding of the potential for different mutations to spread in populations and potentially inform how to exploit fitness costs to combat the spread of resistant strains.

## METHODOLOGY

### Parasite culture

Cryopreserved stocks of *P. falciparum* parasites were thawed and grown in complete media (CM) [RPMI 1640 with L-glutamine (Invitrogen Corp.), 50 mg/l hypoxanthine (Sigma-Aldrich), 25 mM HEPES (Sigma), 0.5% AlbuMAX II (Gibco), 10 mg/l gentamicin (Gibco), and 7.5% NaHCO_3_ (Corning)] at 5% hematocrit using O^+^ RBCs (BioChemed). Cultures were maintained at 37°C in 5% CO_2_, 5% O_2_, and 90% N_2_ and kept below 3% parasitemia with media changes every life cycle (48 h).

Four parasite isolates were cloned and used in pairwise competition assays (Table 1). NF54 is a laboratory-adapted parasite of African origin, MKK2835 is a clinical isolate from the Thai-Myanmar border that was isolated prior to the emergence of ART resistance in the region, NHP4026 is a clinical isolate from the Thai-Myanmar border isolated after the emergence of ART resistance in the region, and NHP1337 is a clinical isolate from the Thai-Myanmar border that was also isolated after the emergence of ART resistance in the region and carries a C580Y *kelch13* mutation. Additional genotypic and phenotypic details are provided in Button-Simons *et al*. [37].

**Table 1:** Parasite isolates competed in pairwise competition assays**.**

Parasite	*kelch13* genotype	ART clearance rate	Parasite clearance half-life (PC_1/2_) [34]	Isolate collection date [34, 35]	Location [34–36]
NF54	Wild-type	Fast	^a^	1979	West Africa
MKK2835	Wild-type	Fast	^a^	2003	Thai-Myanmar border
NHP4026	Wild-type	Slow	8.37	07 December 2007	Thai-Myanmar border
NHP1337	C580Y	Slow	7.84	19 April 2011	Thai-Myanmar border

^a^Parasite clearance half-life (PC_1/2_) was not measured for these parasites

### Life cycle synchronization

Isolates were triple-synchronized using 5% D-sorbitol [38]. The first synchronization occurred when most parasites were at the ring-stage of development; a second synchronization was done 48 h later and a third 8 h after the second. Parasites were considered synchronized when at least 80% of the parasites were rings. Prior to assay initiation, NF54 was 96% rings, NHP4026 was 94%, MKK2835 was 80%, and NHP1337 was 85%.

### Competitive growth assessment of art-resistant and art-sensitive parasites assay setup

Forty-eight hours after the final synchronization, pairwise competitions were set up between four isolates in a 96-well plate [30]. The parasites included two isolates with slow ART clearance rates NHP1337 (C580Y *kelch13* mutation) and NHP4026 (*kelch13* wild-type) and two ART-sensitive isolates MKK2835 and NF54. In this study, we refer to the slow-clearing clinical phenotype, measured as delayed parasite clearance in patients (PC_1/2_ > 5 h), as “ART-resistant,” rather than defining resistance by an *in vitro* drug susceptibility value. Each well contained a total volume of 200 μl, with three technical replicates per competition. Each parasite was set up individually (2% parasitemia) and in their respective mixed competitions (1% each, 2% total parasitemia) to compare growth rates and recovery from drug perturbation. This design kept total initial parasitemia comparable between the individual and mixed parasite competitions and ensured reliable detection of parasites after drug treatment; consequently, per-parasite starting parasitemia was higher in individual cultures than in the competition assay. We aimed to initiate all mixed parasite competitions at equal frequencies of the two strains. Because initial parasitemia is estimated from Giemsa-stained slides, the actual starting ratios deviated from 50:50 in some experiments, as was subsequently quantified by microsatellite genotyping of parasite genomes (described below).

At assay start (0 h), each individual and mixed parasite competition was exposed to 700 nM dihydroartemisinin (DHA; dissolved in dimethyl sulfoxide, DMSO), for 1 and 3 h. Plates were washed three times with incomplete media (ICM; CM without 0.5% AlbuMAX II) to remove the drug. Untreated controls were exposed to DMSO only and were washed with ICM three times. The DHA or mock treatment was repeated at 24 and 48 h for a total of three consecutive days of DHA exposure. No further drug exposures occurred, and parasites were left to recover for the remainder of the assay (64 days total).

Parasitemia for each culture was determined by microscopy (Giemsa-stained slides, 1000 RBCs counted) immediately before each dilution, and samples were collected and stored at −80°C. Fresh media was added every life cycle (48 h), and every other life cycle (96 h), cultures were diluted to ~1% parasitemia with the addition of fresh RBCs and media. Raw parasitemia, dilution-corrected parasitemia, and percent ring-stage counts for each individual parasite and competition can be found in Supplementary Fig. SS1. Every 96 h, aliquots were collected for microsatellite genotyping.

### Amplification and fragment analysis of fluorescent microsatellite markers

Samples collected every other life cycle were used to estimate the relative abundance of each parasite genome in the mixed cultures. Labeled DNA microsatellite markers that yield distinct fragment sizes for each parasite isolate in culture were amplified through polymerase chain reaction (PCR) reactions with the Phusion Blood Direct PCR Kit (Thermo Fisher, cat #F547L, 20 μl reactions) and primers labeled with Well-Red fluorescent dyes (Sigma, custom order) as previously described [30]. Annealing temperatures were determined using the ThermoFisher T_m_ Calculator. Thermocycler conditions were as follows: denaturation for 5 min at 98°C, followed by 30 cycles of 98°C for 1 s, optimal annealing temperature (T_m_) for 5 s (T_m_ = 53°C for the two primers used), and 65°C for 15 s. Final extension was at 65°C for 1 min. Amplified samples were analyzed running fragment analysis using the ABI 3730xI DNA Sequencer (Thermo Fisher).

Fragment analysis data were used to estimate the relative abundance (relative ratio) of each isolate genome in the competition by using the fluorescent peak heights corresponding to the microsatellite size of each isolate as a proportion of the overall signal, as previously described [30]. Two microsatellites were used to differentiate between competitions: TA119 and TA81 (TA119 forward: 5′-TCCTCGATTATATTATTGCA-3′, TA119 reverse: 5′-TAATACATTCCCATTAGATG-3′, TA81 forward: 5′-TGGACAAATGGGAAAGGAT-3′, TA81 reverse: 5′-TTTCACACAACACAGGAT-3′). Resulting proportions from each sampling day (Supplementary Table SS1) were plotted over time.

### Cumulative parasitemia calculation

For each culture, cumulative parasitemia was calculated as the sum of the dilution-corrected parasitemia across all sampling time points. Every 96 h, Giemsa-stained blood smears were made, and parasitemia was counted by microscopy to obtain the raw parasitemia. Parasites were then diluted to ~1% with fresh blood and media. To account for these dilutions, raw parasitemia at each time point was multiplied by the inverse of the dilution factor (e.g. a 50% dilution was corrected by multiplying raw parasitemia by 2; a 75% dilution by 4) before summing over time. For mixed-strain competitions, at each sampling time point, we multiplied the total raw parasitemia by each strain’s relative genome abundance (using microsatellite fragment analysis) to obtain per-strain parasitemia and then corrected these values for the dilution factor at that time point, and then summed the dilution-correct per-strain parasitemia over time to obtain cumulative parasitemia for each strain (Supplementary Table S2).

### Data analysis

Conceptually, our analyses asked two questions (i) does a parasite line produce fewer parasites when grown in competition than when grown alone (competitive suppression), and (ii) is any such suppression alleviated by drug treatment (competitive release)? We addressed these questions by modeling how the relative proportion of each parasite line and cumulative parasitemia change over time under different competition and treatment conditions and then comparing the fitted model trajectories between conditions.

All analyses were performed in RStudio (2025.09.1 + 401) using tidyverse, readxl, mgcv, and gratia packages; the entire pipeline is available on github (https://github.com/FerdigLab/Competitive-Suppression-and-Release) with documentation. For each parasite pair and treatment condition, we modeled the temporal dynamics of proportions using generalized additive models (GAMs). Models included organism-specific smooth functions of time and, when available, random intercepts for the replicate experiments. To quantify competitive differences over time, we computed pairwise contrasts between organisms on the linear-predictor scale using either simulation-based smooth differences or equivalent matrix-based contrasts derived from the model coefficients and covariance structure. Time points at which the 99.9% confidence interval (CI) for the contrast excluded zero were considered statistically significant, and sustained divergence was defined as ≥4 consecutive significant days on a sampling point.

For each strain × treatment combination, cumulative parasitemia trajectories were analyzed using GAMs with a negative binomial model. Fitted means on the response scale (“μ/mu”) were obtained for both contexts. To assess the effect of competition, we computed a valid time-varying contrast of the linear predictor (together vs. alone), with 99.9% CIs derived analytically from the model’s covariance matrix. In addition, for each organism × context (alone or together) combination with both UT and T available, we fitted negative binomial GAMs and computed time-varying contrasts of the linear predictor for treated vs. untreated (treatment effect) using the same 99.9% CIs and sustained-divergence criterion. Finally, for each competition pair and treatment, we modeled the cumulative parasitemia of both parasite lines jointly and obtained time-varying contrasts of the linear predictor comparing the two organisms within the same competition (e.g. cumulative NF54 vs. cumulative NHP4026 in NF54/NHP4026 co-cultures). For both datasets, we recorded daily significance indicators, the earliest day of sustained statistical divergence (“first significant day”), and model-based fitted trajectories and contrast curves. All analyses were restricted to Days 0–64 (Supplementary Tables S3–S10).

## RESULTS

### The length of DHA drug exposure does not impact final treated competitive outcomes

**Figure 2:**
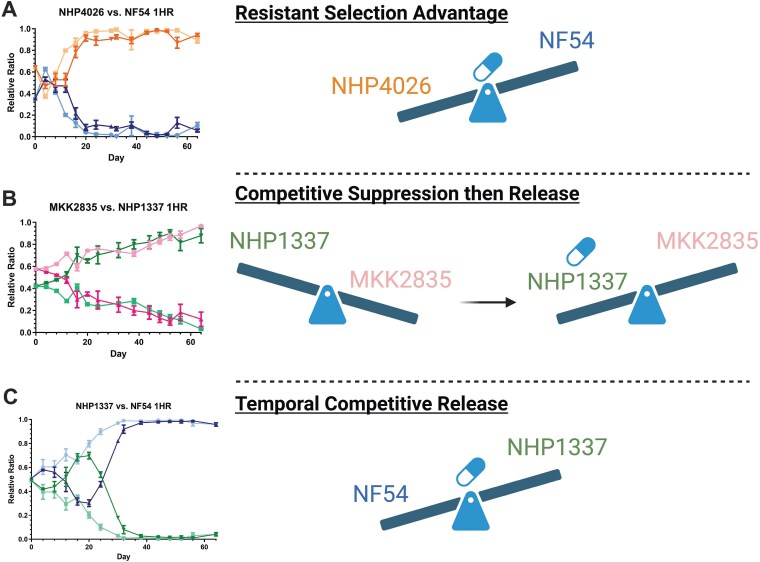
**Competitive growth outcomes with a 1-h, 3**-**day DHA exposure**; each competition was either exposed to DHA for 1 h for three consecutive days (Days 0, 1, 2) (dark colors, triangles) or was treated with DMSO (untreated, light colors, circles and squares); (A) NHP4026 (ART-R, orange) outcompetes NF54 (ART-S, blue) in both the treated and untreated competitions; (B) MKK2835 (ART-S, red) outcompetes NHP1337 (ART-R, green) in the untreated competition, but loses the treated competition; (C) NF54 (ART-S, blue) eventually outcompetes NHP1337 (ART-R, green) in both the treated and untreated competitions.

Two drug exposures were used for treatment of the four competitions over three consecutive days: 700 nM DHA for 1 h or 700 nM for 3 h. Notably, the same competitive outcome was seen regardless of the length of DHA exposure (Fig. 2 and Supplementary Fig. S2). Therefore, all further analysis focused on the 1-h drug exposure experiment. Parasitemia was graphed as cumulative parasitemia, defined as the sum of dilution-corrected parasitemia across the duration of the assay (see METHODS). This metric captures the total number of infected RBCs produced over time, and for competitions, we calculated strain-specific cumulative parasitemia by weighting the dilution-corrected parasitemia at each time point by each strain’s relative genome abundance from microsatellite genotyping and then summing over time.

### Sensitive strain (NF54) is competitively suppressed by resistant strain (NHP4026)

**Figure 3:**
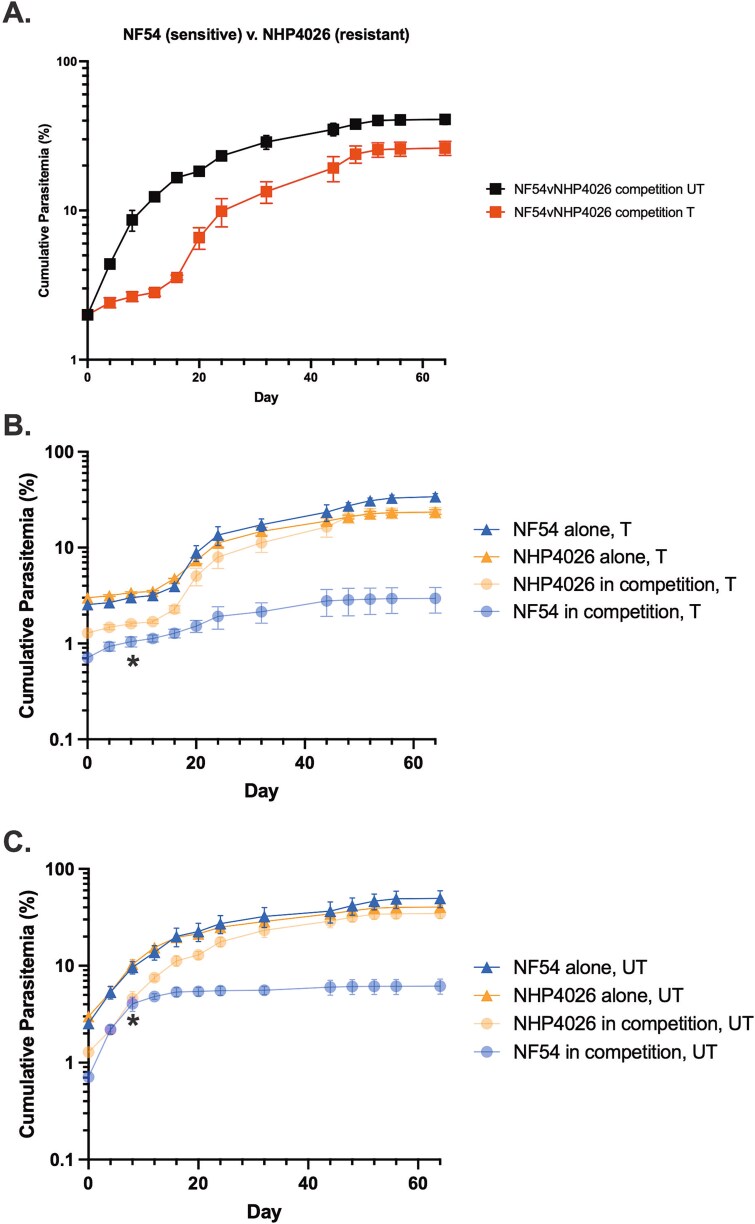
**Growth dynamics of treated and untreated NF54 (sensitive) and NHP4026 (resistant) isolates grown alone (triangles) and in competition (circles)**; (A) cumulative parasitemia for treated and untreated NF54 vs. NHP4026 competitions, showing drug-induced reduction in growth from Day 0 to 16; (B) cumulative parasitemia for competitors grown individually (triangles) and competitors grown in competition (circles) in DHA-treated competition, showing the parasitemia of NHP4026 became significantly different (estimate = −1.08, 99.9% CI: −1.82 to −0.34) at Day 8^*^, when the parasitemia of NF54 in competition plateaued; (C) untreated competition dynamics show the parasitemia of NF54 plateaued only when in competition, beginning on Day 8^*^ (estimate = −1.01, 99.9% CI: −1.73 to −0.29). Asterisk (^*^) corresponds with first day of significance.

The NF54 versus NHP4026 competition resulted in NHP4026 outcompeting NF54 both with and without drug pressure (Fig. 2a). To assess the growth dynamics of NF54 (ART-S) against NHP4026 (ART-R) after drug treatment, the cumulative parasitemia of each competitor was tracked individually and in competition. The treated and untreated competitions show similar trends, with a steady increase in cumulative parasitemia after treated parasites recover on Day 16 (Fig. 3a). With DHA pressure, NHP4026 in competition (Fig. 3b, orange circles) displayed similar cumulative parasitemia to NHP4026 and NF54 grown alone, indicating a competitive advantage in recovery from DHA treatment relative to NF54 in competition. In untreated competitions, both isolates maintained stable parasitemia until the parasitemia of each isolate became significantly different (estimate = −1.08, 99.9% CI: −1.82 to −0.34) at Day 8, when NF54 in competition plateaued, displaying suppression by NHP4026 (Fig. 3c). Comparing these competitions with isolates grown alone (Fig. 3b and c, triangles, comparison in Supplementary Fig. S3a) shows that the plateau of NF54 is unique to its competition with NHP4026, as NF54 maintains stable parasitemia when grown alone with or without DHA. This suggests that NHP4026 suppresses NF54 regardless of drug presence. The more resistant NHP4026 outcompetes NF54 under all conditions, possibly due to intrinsic growth advantages that are not solely dependent on drug pressure. Understanding these competitive interactions is crucial for interpreting how ART-resistant and sensitive parasites behave under treatment and within mixed infections.

### Resistant strain (NHP1337) is competitively suppressed by sensitive strain (MKK2835) without drug pressure, but is competitively released with drug pressure

**Figure 4:**
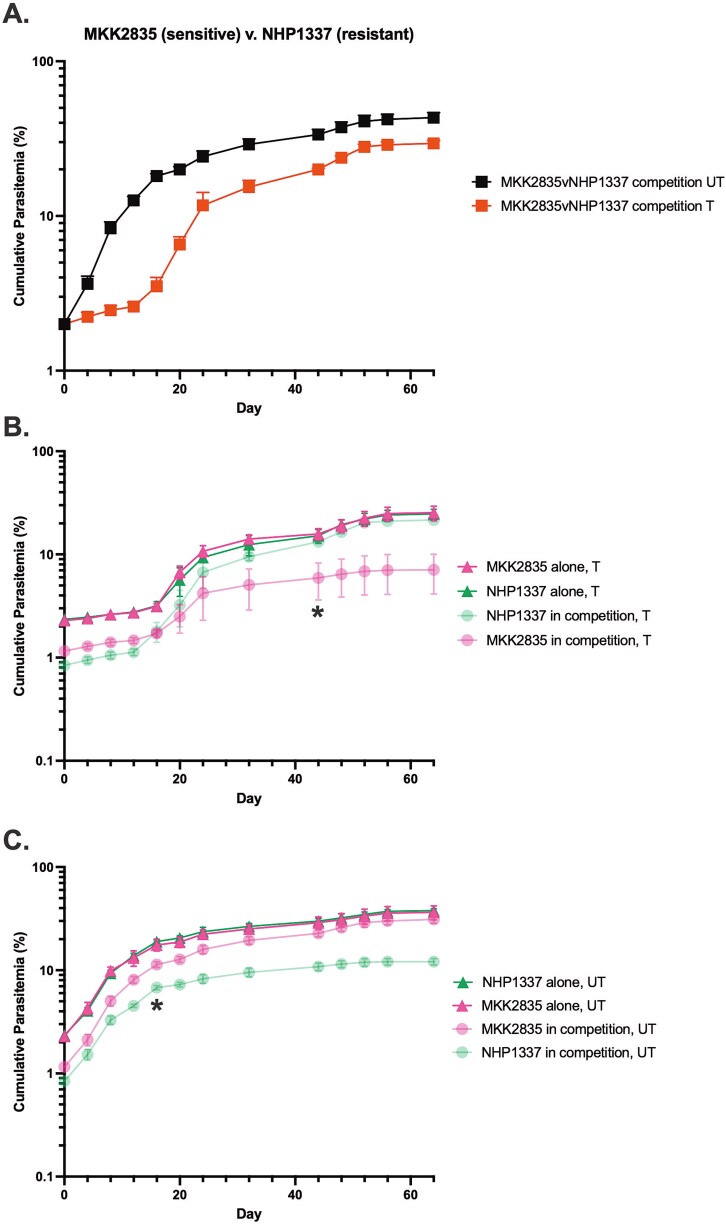
**Growth dynamics of treated and untreated MKK2835 (sensitive) and NHP1337 (resistant) isolates as individuals (triangles) and in competition (circles)**; (A) cumulative parasitemia for treated and untreated MKK2835 vs. NHP1337 competitions, showing drug-induced reduction in parasitemia from Day 0 to 16; (B) cumulative parasitemia for the treated competition; growth of MKK2835 in competition remains stable until Day 44^*^ (estimate = −0.94, 99.9% CI: −1.70 to −0.18), while NHP1337 recovers more quickly and continues growing at a rate similar to parasites grown individually; (C) cumulative parasitemia for the untreated competition shows that growth of MKK2835 in competition remains stable, while growth of NHP1337 in competition plateaus with MKK2835 having a significant trajectory by Day 16^*^ (estimate = 0.59, 99.9% CI: 0.11 to 1.07); notably, as individuals, MKK2835 and NHP1337 with and without DHA grow at a relatively stable rate throughout the competition. Asterisk (^*^) corresponds with first day of significance.

The MKK2835 versus NHP1337 competition resulted in different outcomes depending on drug treatment (Fig. 2b). MKK2835 (ART-S) outcompeted NHP1337 (ART-R) in the absence of drug, indicating a fitness cost for resistance. However, under drug treatment, NHP1337 dominated, suggesting competitive release due to its resistance (Fig. 4). In the treated competition, the parasitemia of NHP1337 recovered and was significantly different (estimate = −0.62, 99.9% CI: −1.00 to −0.24) by Day 16 and maintained steady growth, while MKK2835 recovered on Day 20 and grew at a slower rate relative to NHP1337 (Fig. 4b, circles). In contrast, the untreated competition showed stable parasitemia for MKK2835, while NHP1337 exhibited a plateau in parasitemia and grew at a slower rate relative to MKK2835 (Fig. 4c, circles, comparison in Supplementary Fig. S3B), suggesting suppression of NHP1337 by MKK2835. Models predict a significant trajectory between the parasitemias of the two parasites by Day 4 in the untreated competition (estimate = 0.82, 99.9% CI: 0.37 to 1.27), MKK2835 established dominance in the absence of drug, while NHP1337 took over under drug pressure, demonstrating competitive suppression and release depending on the presence or absence of drug.

### Resistant parasite (NHP1337) is competitively suppressed by sensitive strain (NF54)

The NF54 versus NHP1337 DHA-treated competition revealed a dynamic interplay between sensitive and resistant parasites, with competitive release only observed in the first 24 days, after which NF54 began to suppress NHP1337 and ultimately emerged as the “winner” of the competition (Fig. 2c). Initially, NHP1337 displayed a brief period of competitive release, likely due to its resistance-associated adaptation allowing it to outgrow NF54 temporarily. However, by Day 16, NF54 began to recover, steadily increasing its parasitemia while the growth rate of NHP1337 declined around Day 24 (Supplementary Fig. S4B). This suppression of NHP1337 was established around Day 24 (estimate = 1.30, 99.9% CI: 0.33 to 2.26) and maintained throughout the rest of the competition, indicating that NF54, despite being drug-sensitive, may possess an inherent competitive advantage, possibly because NF54 is a commonly used laboratory strain, whereas NHP1337 is a more recently adapted patient isolate. Comparing these results to the performance of NHP1337 in monoculture confirms that the growth rate decline is specific to the competitive setting, as NHP1337 remained stable when grown alone with and without DHA pressure (Supplementary Fig. S4B and S4C, comparison in Supplementary Fig. S3C). This suggests that NF54 might outcompete NHP1337 through resource competition or other growth mechanisms.

## DISCUSSION

Fitness costs have been associated with ART-resistance, but the genetic complexity underlying resistance and its impact on fitness without drug remain incompletely understood [25, 28–30]. Drug exposure, along with various host factors such as immunity and nutrients, strongly influences *P. falciparum* infection outcomes [20]. The *in vitro* competitive growth assay with clinically relevant drug treatment described here can be used to model how co-infecting drug-sensitive and drug-resistant parasites are impacted by drug pressure.

ART-resistant parasites first emerged and spread in Southeast Asia, an area with low parasite transmission, low COI, and extensive antimalarial drug treatment [3]. Several theories aspire to explain the emergence and spread of ART-resistance, including the intricate balance of fitness costs and drug pressure on the malaria parasite. A common presumption, in both theoretical and empirical work, is that without compensatory mutations, drug resistance mutations impose a fitness cost in the absence of drug, resulting in competitive suppression and, when drugs are introduced, competitive release of the resistant parasite. Under this view, sensitive parasites should outcompete resistant parasites without drug (competitive suppression), whereas drug treatment should reverse this disadvantage and allow resistant parasites to outcompete sensitive parasites (competitive release).

However, fitness costs of antimalarial resistance are not a given: their presence and magnitude can vary across genetic backgrounds and ecological contexts, and compensatory changes can mitigate or erase initial fitness costs (as reviewed in [16]). Our results also support this more nuanced picture. Previously, there has been evidence of competitive release in rodent malaria models [24, 25, 39, 40] and evidence of competitive release using an *in vitro P. falciparum* model with lab lines [23]. To our knowledge, our study is the first to test for competitive suppression and release using recent *P. falciparum* patient isolates in an *in vitro* system that directly compares co-cultures and monocultures grown with and without drug. Using clinical isolates, we defined ART-resistance as the slow-clearing clinical phenotype (delayed parasite clearance) rather than a potentially less relevant *in vitro* susceptibility measure. Fitness costs associated with this slow-clearing phenotype may differ from those associated with other resistance phenotypes: adaptations that prolong parasite clearance in patients could have relatively small effects on asexual growth *in vitro*, whereas some resistance mechanisms more directly impair growth in the absence of drug.

Four isolates were used in three unique competitions in the absence and presence of DHA: NHP4026 (ART-R) versus NF54 (ART-S), NHP1337 (ART-R) versus MKK2835 (ART-S), and NF54 (ART-S), versus NHP1337 (ART-R). Given the classic competitive suppression and release hypothesis, we predict that in the absence of DHA, the ART-sensitive isolates (NF54, MKK2835) would outcompete the ART-resistant isolates (NHP4026, NHP1337), and that this pattern would reverse under DHA treatment. However, the assumption of a greater fitness cost to being more drug-resistant does not always hold true and we observed three distinct outcomes. In the NHP4026 versus NF54 competition, the more ART-resistant parasite (NHP4026) outcompeted the more ART-sensitive parasite (NF54) both with and without DHA (Fig. 2a). In the NF54 versus NHP1337 competition, NF54 (ART-S) outcompeted NHP1337 (ART-R) with and without DHA (Fig. 2c). Only in the NHP1337 versus MKK2835 competition did we observe the expected pattern of competitive suppression without DHA and competitive release with DHA. (Fig. 2b). In summary, among the three competition pairs, the classical expectation held in only one case, demonstrating that a greater fitness cost does not always scale with drug-resistance.

These patterns can be interpreted in at least two ways. First, fitness costs to the resistant parasite may be minimal or absent in some genetic backgrounds, especially if selection has favored variants that mitigate any initial fitness-resistance trade-offs. Second, our *in vitro* system may not capture key aspects of within-host biology (immune responses, sequestration, resource limitation) that could reveal fitness costs *in vivo*. Distinguishing between these explanations will require integrating *in vitro* competition assay data with *in vivo* and epidemiological data. The lack of a consistent fitness cost and the strain-specific outcomes we observe suggest that predictions about the spread of ART resistance cannot rely on the assumption that resistant parasites will be automatically outcompeted without drug pressure.

Recovery from drug can differ between isolates in competition [41], which could be clinically impactful in the case of the MKK2835 versus NHP1337 competition. Without DHA, MKK2835 (ART-S *kelch13-*WT) outcompeted NHP1337 (ART-R *kelch13*-C580Y), but in the presence of DHA, NHP1337 outcompeted MKK2835. Because these isolates came from the same geographic area, a co-infection treated with an ART derivative could preferentially transmit the *kelch13*-C580Y parasite, a potential worst-case scenario of competitive release enhancing the spread of resistance. It is now well established that multidrug-resistant KEL1/PLA1 lineage parasites with *kelch13*-C580Y mutations have swept Southeast Asia and spread in the region, suggesting that they have a fitness advantage over other parasites [42]. As many countries move toward malaria elimination, choices of chemopreventives and front-line therapies will be critical for balancing selection for drug resistance and effective malaria control [24, 39].

Together with other recent work [16, 25, 28–30], our findings reinforce that fitness consequences of resistance are highly context dependent and can only be understood by directly measuring competition across diverse parasite genetic backgrounds and drug regimens. Our study did not look at gametocyte production; however, it has been shown in *Plasmodium chabaudi* that in the presence of a competitor, a resistant clone produced more blood stage parasites and gametocytes compared with the absence of a competitor [24]. Although *in vitro* models cannot replicate host immunity, nutrient status, or pharmacodynamics, they can shed light on important questions about basic parasite growth dynamics: Can parasites recover more easily in a co-culture than in a monoculture? Can parasites sense each other [15, 43]? How do parasites balance competitive success with virulence and host survival? The competitive assays used here are a first step toward quantifying these dynamics and can be extended to diverse panels of *P. falciparum* isolates and alternative drug combinations to better predict which resistant genotypes are most likely to spread.

## CONCLUSIONS AND IMPLICATIONS

Our three competitions establish an *in vitro* drug treatment protocol that can be applied to future competitions between relevant field isolates. Including side-by-side monocultures along with co-cultures was informative to investigate the impact of competitors on parasite growth. Importantly, the assumption that there is a greater fitness cost to being more drug-resistant is not always the case. Modeling competitive suppression and release with patient-derived isolates can clarify how the fitness costs of ART-resistance depend on drug selection and will help predict which resistant genotypes are most likely to spread, informing strategies to exploit fitness costs to limit resistance.

## Supplementary Material

Supplementary_material_eoag009

## Data Availability

The data underlying this article are available in the article and in its online supplementary material.
