# Supplementary material for: Competitive suppression and release in artemisinin-resistant Plasmodium falciparum field isolates
Source: Evol Med Public Health. 2026 May 7;14(1):1–12. doi: 10.1093/emph/eoag009 (PMC13271393; doi:10.1093/emph/eoag009)
Supplement: Supplementary_material_eoag009 [file supplementary_material_eoag009.zip › New Microsoft Word Document.docx]

**Supplemental table 1.** Relative ratios (via fragment analysis) of each of the competitors throughout the three competitions (NF54 v. NHP4026, MKK2835 v. NHP1337, and NF54 v. NHP1337) for 1 h and 3 h treatments.

**Supplemental table 2.** Cumulative parasitemias (counted via Giemsa-stained slides and adjusted for parasitemia dilutions) throughout the 64-day period for each parasite grown alone and in competition, both UT and T (three technical replicates per condition). Raw parasitemia, dilution factors, and dilution-corrected parasitemia are also included.

**Supplemental table 3.** Point-estimate summaries for the treated vs. untreated cumulative parasitemia contrasts.

**Supplemental table 4.** Point-estimate summaries for cumulative parasitemia contrasts (together vs. alone), reporting the contrast and 99.9% CI at the earliest day of sustained significance.

**Supplemental table 5.** Point-estimate summaries for organism-vs-organism cumulative parasitemia contrasts within each competition.

**Supplemental table 6.** Point-estimate summaries for each competition contrast, reporting the contrast estimate and 99.9% CI at the earliest day of sustained divergence.

**Supplemental table 7.** Daily linear-predictor contrasts for each competition (organism A – organism B) with 99.9% confidence intervals and significance indicators across time.

**Supplemental table 8.** Daily cumulative parasitemia contrasts between organisms within the same competition (e.g., NF54 cumulative vs. NHP4026 cumulative), with 99.9% CIs and significance indicators.

**Supplemental table 9.** Daily treatment contrasts for cumulative parasitemia (T – UT) within each organism × context combination, with 99.9% confidence intervals and significance indicators.

**Supplemental table 10.** Daily linear-predictor contrasts of cumulative parasitemia for each organism (together – alone) with 99.9% confidence intervals and significance indicators across time.
