# Supplementary material for: Competitive suppression and release in artemisinin-resistant Plasmodium falciparum field isolates
Source: Evol Med Public Health. 2026 May 7;14(1):1–12. doi: 10.1093/emph/eoag009 (PMC13271393; doi:10.1093/emph/eoag009)
Supplement: Supplementary_material_eoag009 [file supplementary_material_eoag009.zip › Supplemental Figures.docx]

**SUPPLEMENTARY INFORMATION**


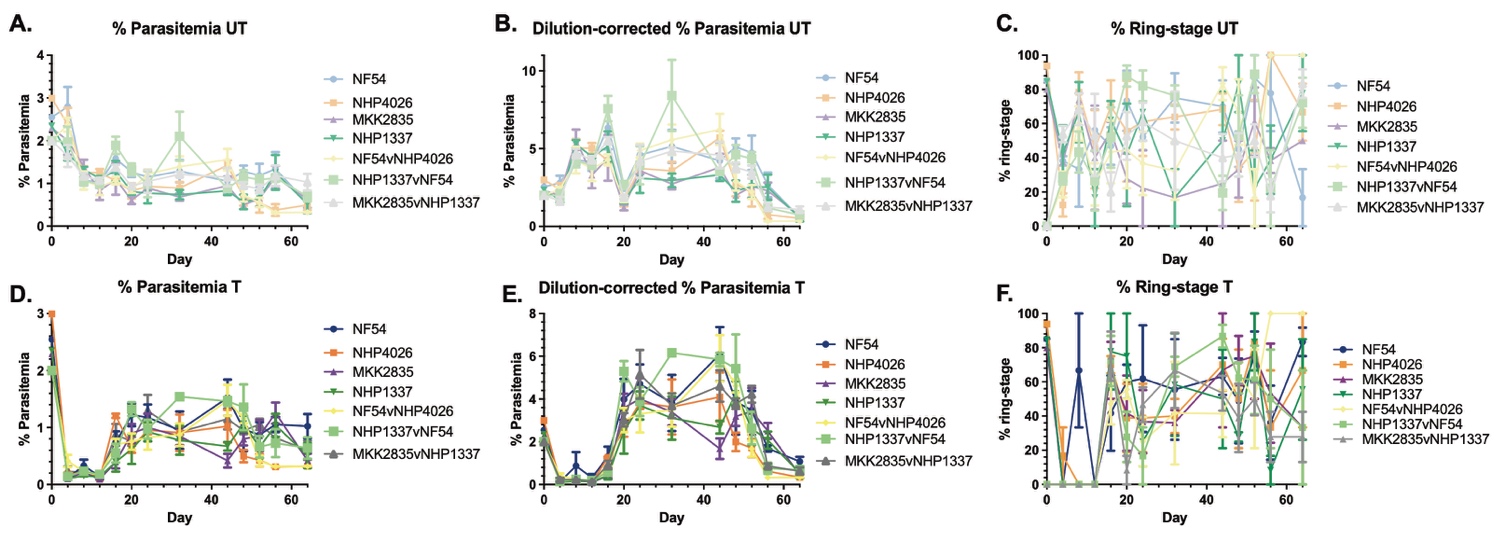


**Supplemental Figure 1. Raw percent parasitemia and percent ring-stage counts for each individual parasite and each competition with and without drug treatment.** (A) Raw percent parasitemia for parasites grown alone and in competition without drug treatment (UT). (B) Dilution-corrected percent parasitemia for the same cultures shown in panel A. (C) Percent ring-stage parasites (percentage of all parasites at ring-stage). (D) Percent parasitemia for each parasite grown alone and in competition with drug treatment (T). (E) Dilution-corrected percent parasitemia for the same cultures shown in panel D. (F) Percent ring-stage parasites for all T parasites grown alone and in competition. These percent parasitemia plots illustrate the underlying data used to calculate cumulative parasitemia.


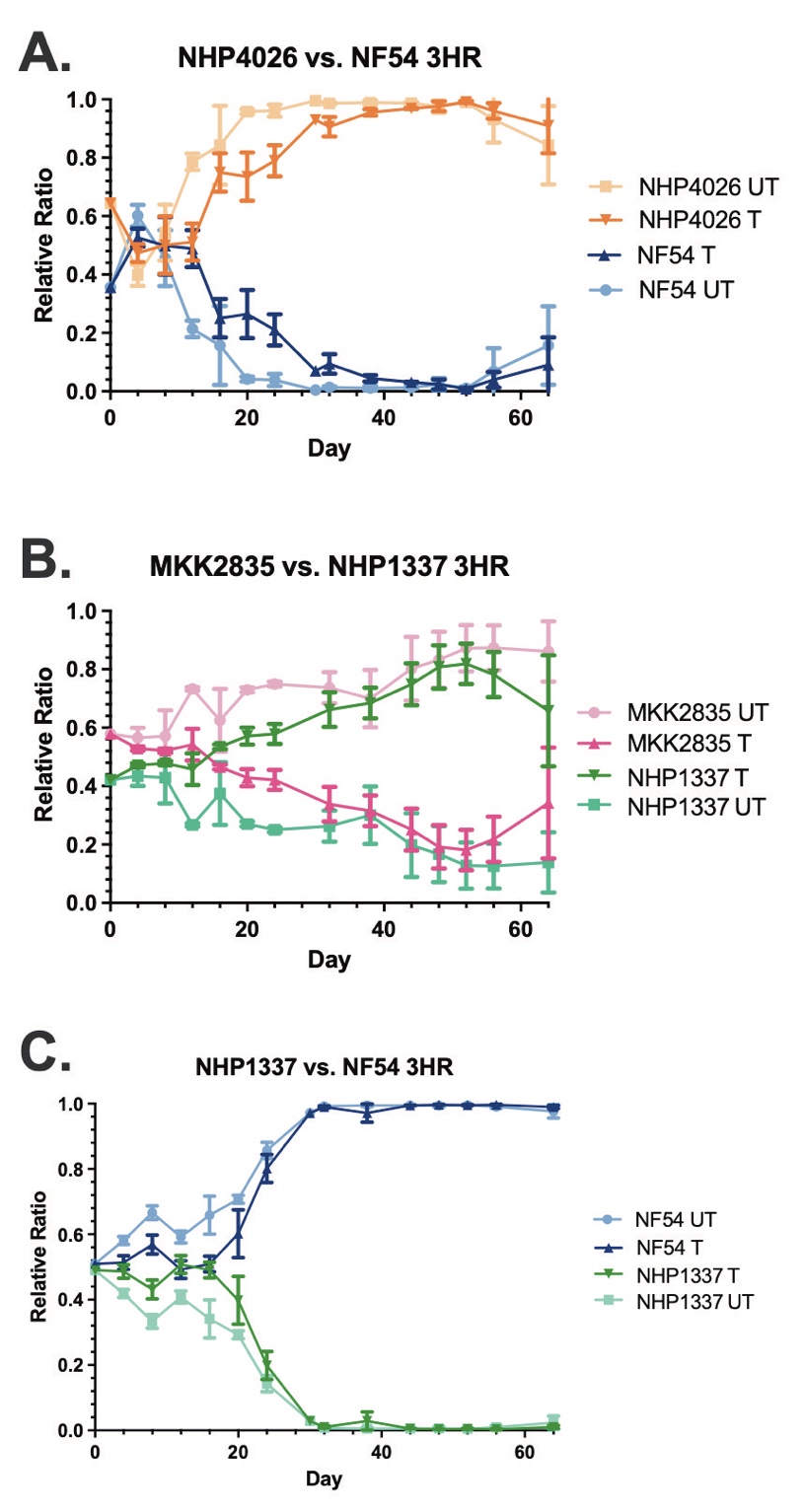


**Supplemental Figure 2. Competitive growth outcomes with 3-hour, 3 day drug exposure between four isolates: (A) NHP4026 vs. NF54, (B) KK2835 vs. NHP1337, and (C) NHP1337 vs. NF54.** Each competition was exposed to a 3-hour pulse of 700 nM DHA for three consecutive days (day 0, 1, 2). Each drug exposure is graphed with its corresponding DMSO UT isolate competitions. While recovery times differed slightly for the different drug exposures, the overall outcome of the competitions by day 64 were the same as from Figure 2 (1-hour, 3 day exposure).


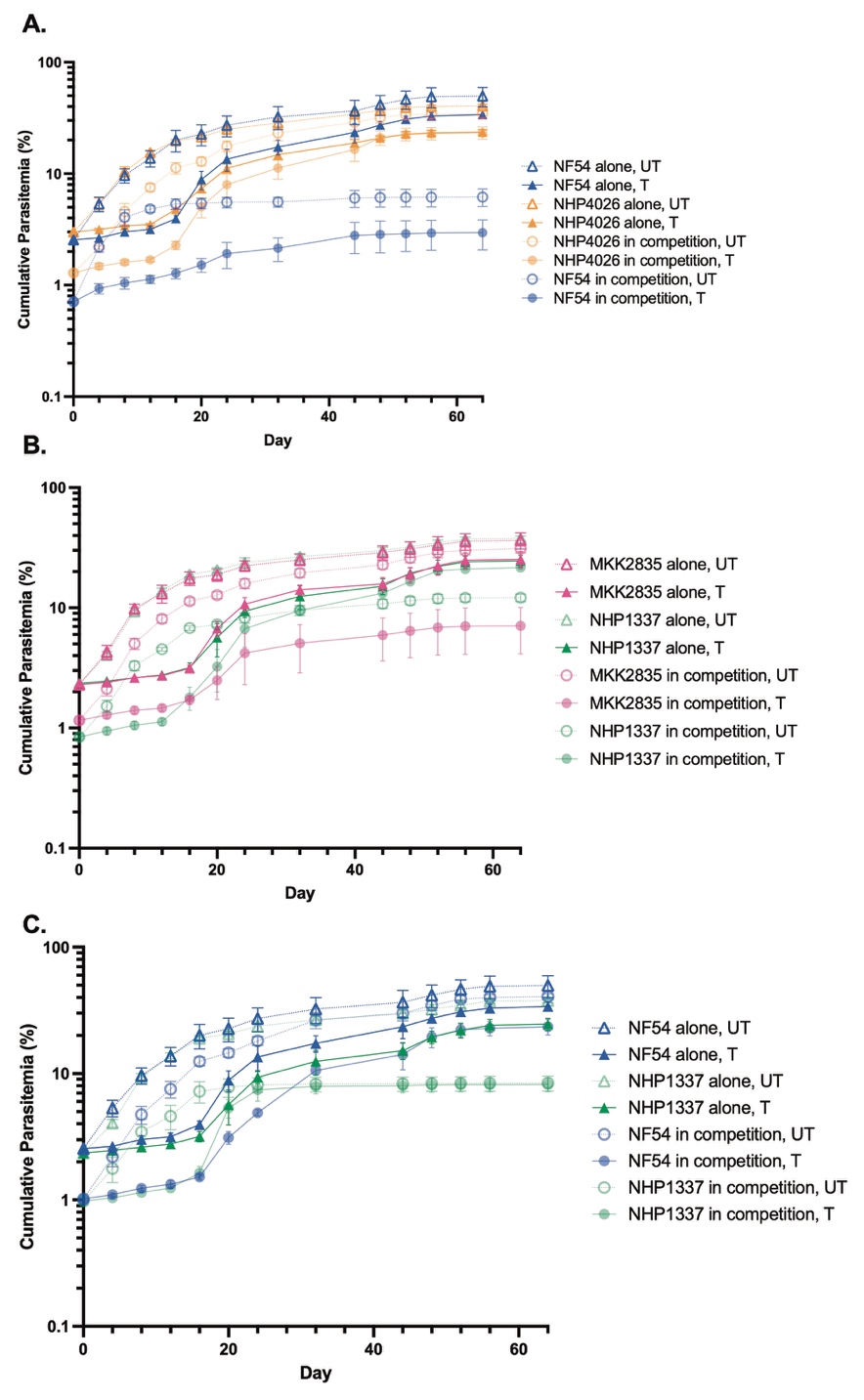


**Supplemental Figure 3. Growth dynamics of treated and untreated parasites grown alone and in competition.** Cumulative parasitemia are shown for parasites growth alone (triangles) and in competition (circles) under untreated (UT) and DHA-treated (T) conditions. (A) NF54 vs. NHP4026 competitions and parasites grown alone (same data as Figure 3B and C). (B) MKK2835 vs. NHP1337 competitions and parasites grown alone (same data as Figure 4B and C). (C) NF54 vs. NHP1337 competitions and parasites grown alone (same data as Supplemental Figure 4B and C).


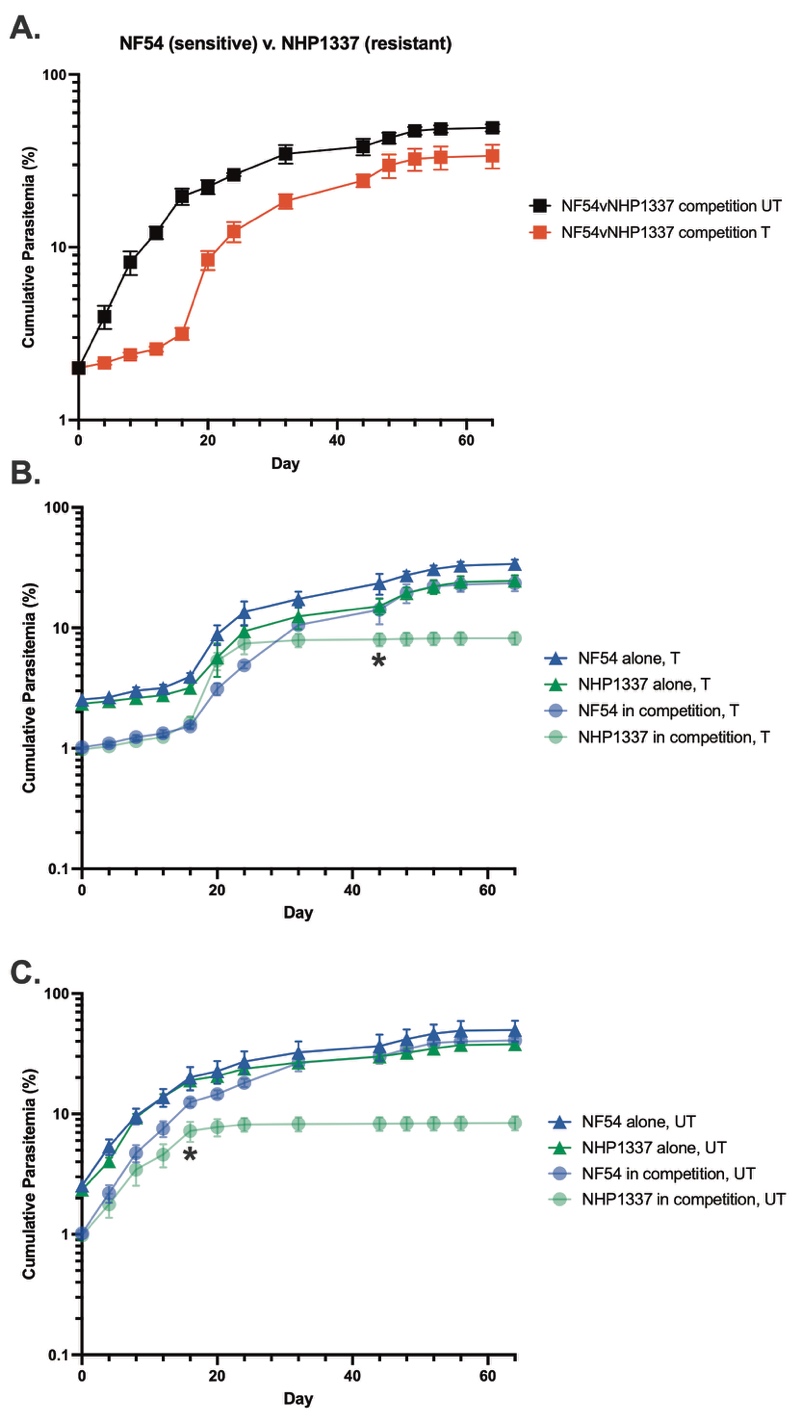


**Supplemental Figure 4.** **Growth dynamics of treated and untreated NF54 and NHP1337 grown alone (triangles) and in competition (circles).** (A) Cumulative parasitemia for treated (red) and untreated (black) NF54 vs. NHP1337 competitions, showing drug-induced reduction in parasitemia from day 0 to 16. (B) Cumulative parasitemia of the treated competitions and parasites grown alone show the decrease in growth rate for NHP1337 in competition around day 44* (estimate = 0.66, 99.9% CI: 0.15 to 1.18), which is when the suppression of NHP1337 by NF54 begins. NHP1337 alone grows consistently throughout the duration of the treated assay. (C) Cumulative parasitemia for the untreated competition and parasites grown alone also show the decreasing growth rate of NHP1337 in competition around day 16* (estimate = 0.58, 99.9% CI: 0.07 to 1.09); NHP1337 is outcompeted by NF54 without DHA treatment. Asterisk (*) corresponds with first day of significance.
